# The I. H. A. and Its Work

**Published:** 1883-12

**Authors:** 


### HOMŒOPATHIC PHYSICIAN.

A MONTHLY JOURNAL OF MEDICAL SCIENCE.

If our school ever gives up the strict inductive method of Hahnemann, we are lost, and deserve only to be mentioned as a caricature, in the history of medicine."—CONSTANTINE HERING.

Vol. III. DECEMBER, 1883. No. 12.

### EDITORIAL.

THE I. H. A. AND ITS WORK.—There are useful societies and useless societies; there are societies which are only ornamental and societies which work industriously. In which of these classes shall the I. H. A. be placed? At the present time it can scarcely be honestly classed among the societies that perform useful work. Apathy and indifference, rather than interest and zeal, characterize its membership. If the members of this Association are satisfied with the work they have accomplished in the past, then they are very easily pleased. Let any member glance over the transactions of its past sessions to discover, if he can, wherein Homœopathy has been benefited by the existence of the I. H. A. Resolutions innumerable have been introduced; clinical cases, numerous and interesting, have been read. The former are mostly useless, and often very silly; the latter have been useful and valuable to a certain extent, but they present little that is new. Let this Association now show by work—original and real, not by *talk*—what it professes and practices.

As we understand it, this Association was organized for the purpose of advocating and upholding these three cardinal principles of Homœpathy:

1st. The Law of the Similars: the totality of the symptoms the only guide for prescribing.

2d. The Single Remedy: in contradistinction to compound prescriptions, alternation, and the use of adjuvants, such as tonics, narcotics, etc.

3d. The Minimum Dose: that is, a dose sufficient to cure, not to aggravate.*

These being acknowledged as an epitome of our principles—our platform, so to speak—it is our duty not only to advocate, support, and elucidate them, but it is even more a duty to facilitate in every way possible their application in practice. Every weak point should be strengthened; every deficient instrument, supplied. If our therapeutics need revising, it should be done; if a new work be needed, it should be supplied. And no one can deny that such needs do exist. Let the I. H. A., then, be about its proper work. Let it undertake such work as is being done by the Sydenham Society and by the Hahnemann Publishing Society, both of England. They are publishing standard works—such works as are useful and necessary, but would not pay a publisher to bring out. Let the I. H. A. do likewise.

In default of a better plan, we would suggest that its Bureau of Materia Medica be appointed to this work. Let the Association at each session choose a subject upon which a practical work is needed and let its committee edit and publish this during the following year, or as soon after as practicable. The Association to pay the cost

* "The corollary, *the minimum dose of the dynamized remedy*, though not made a necessity by the logical requirements of the law, is fully sustained by reason and the records of long practical experience. By the minimum dose, we understand the least quantity of the drug the cure requires. As the object for which it is given is the cure, all that is given more than this requires is always superfluous, to say the least, and may be—and often is—a source of mischief; therefore reason sanctions and requires this minimum. That the dose be not only the least that will effect the cure, but that it be also a potentized dose, it is believed is required in order to the realization of the best practical results from the administration of our therapeutic law. This is accepted on the testimony of those who have tried it extensively and compared the results of the use of potentized and unpotentized drugs. They say their experience fully sustains the greater curative power of the former. It is self-evident the value of this corollary must be decided by this practical tribunal, *i. e.*, by the experience of those who have practically tried both the dynamized and crude drug and compared the results of the use of each. It is just here that *a priori* reasoning of those who have made no such trial has no place, and cannot, with reasonable men, have the least weight as against the testimony of those who have."—P. P. WELLS.

of publishing, each member to receive a copy, outsiders to pay extra for them.

Many such works are needed. Menstruation and its concomitant troubles might serve for one year, the heart and its troubles for another, and so on. By such work the Association would *interest* and improve its members, would advance Homœopathy, and would shortly command the respect of the homœopathic world. Shall we do it?

This article is offered merely as a suggestion. If others have a better plan of work, or improvements on the one we offer, we should be glad to hear from them.

